# Supplementary material for: Remarkable flexibility in freestanding single-crystalline antiferroelectric PbZrO3 membranes
Source: Nat Commun. 2024 May 24;15:4414. doi: 10.1038/s41467-024-47419-w (PMC11116490; doi:10.1038/s41467-024-47419-w)
Supplement: Supplementary file 1 — Supplementary Information [file 41467_2024_47419_MOESM1_ESM.pdf]

Supplementary Information

Remarkable Flexibility in Freestanding Single-crystalline Antiferroelectric $PbZrO_3$ Membranes

Yunting Guo[1†], Bin Peng[1†,], Guangming Lu[2,3†], Guohua Dong[1], Guannan Yang[1], Bohan Chen[1], Ruibin Qiu[1], Haixia Liu[1], Butong Zhang[1], Yufei Yao[1], Yanan Zhao[1], Suzhi Li[3]*, Xiangdong Ding[3], Jun Sun[3], Ming Liu[1]**

[1] State Key Laboratory for Manufacturing Systems Engineering, Electronic Materials Research Laboratory, Key Laboratory of the Ministry of Education, School of Electronic Science and Engineering, Xi'an Jiaotong University, Xi'an 710049, China

[2] School of Environmental and Material Engineering, Yantai University, Yantai 264005, China

[3] State Key Laboratory for Mechanical Behavior of Materials, Xi'an Jiaotong University, Xi’an 710049, China

* Corresponding author: pengbin@xjtu.edu.cn, lisuzhi@xjtu.edu.cn, mingliu@xjtu.edu.cn

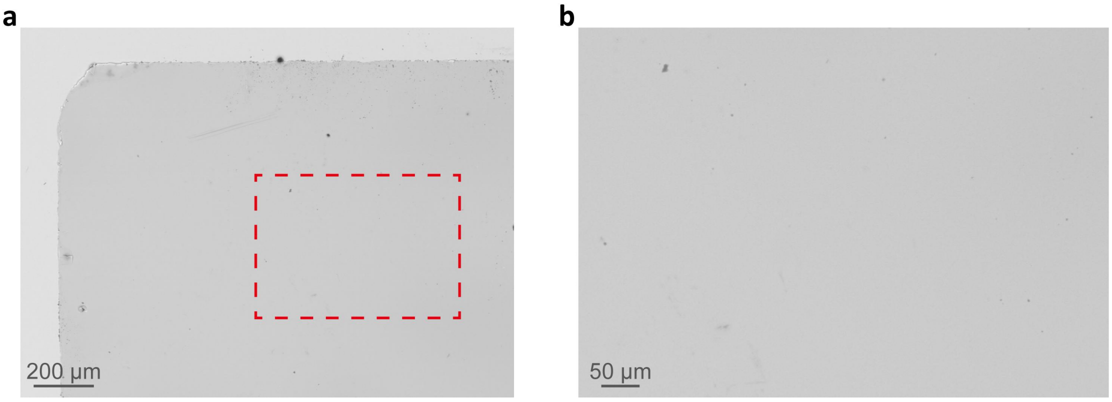

Fig. S1 a-b Scanning electron microscope (SEM) images of transferred freestanding single-crystalline $PbZrO_3$ membrane (a) and corresponding partially enlarged view (b).

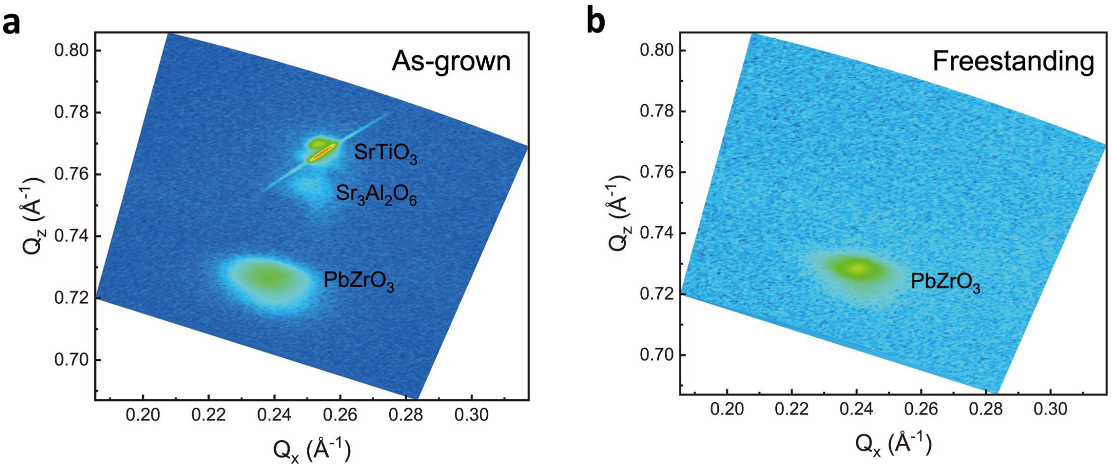

Fig. S2 a-b Reciprocal space mapping (RSM) studies of $PbZrO_3$ film around (103) peak before (a) and after (b) releasing from the substrate.

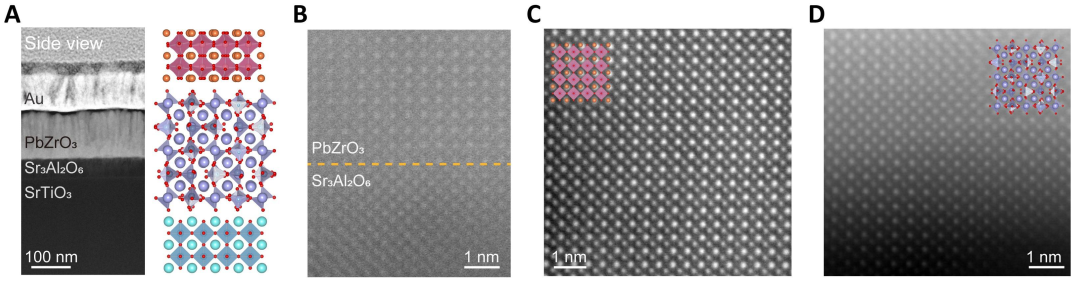

Fig. S3 Microstructure of as-grown $SrTiO_3/Sr_3Al_2O_6/PbZrO_3$ heterostructure. **a** Cross-sectional TEM image of a $SrTiO_3/Sr_3Al_2O_6/PbZrO_3$ heterostructure. **b-d** Cross-sectional HAADF-STEM image of (b) the interface between $Sr_3Al_2O_6$ and $PbZrO_3$, (c) $PbZrO_3$ layer and (d) $Sr_3Al_2O_6$ layer.

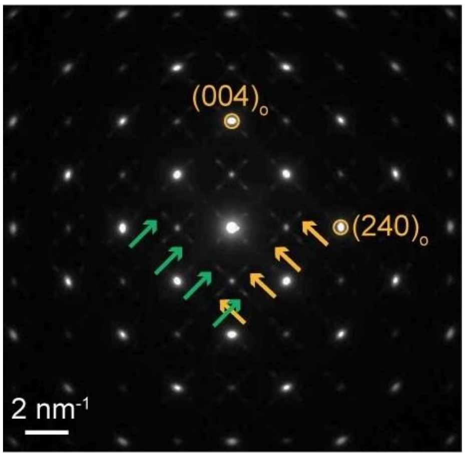

Fig. S4 The selected-area electron diffraction pattern of freestanding $PbZrO_3$ membrane.

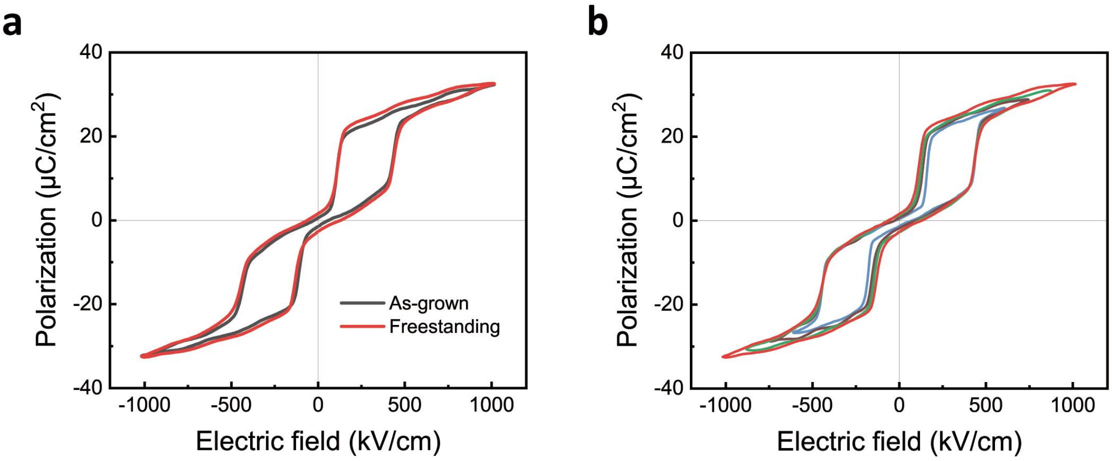

Fig. S5 The P-E hysteresis loops at higher applied electric field. **a** The *P-E* hysteresis loops at higher applied electric field of as-grown $SrTiO_3$/$Sr_3Al_2O_6$/$SrRuO_3$/$PbZrO_3$ heterostructure and freestanding $SrRuO_3$/$PbZrO_3$ membranes on the indium tin oxide (ITO) coated PET substrate. **b** The *P-E* hysteresis loops of freestanding $SrRuO_3$/$PbZrO_3$ membranes with the increasing electric field.

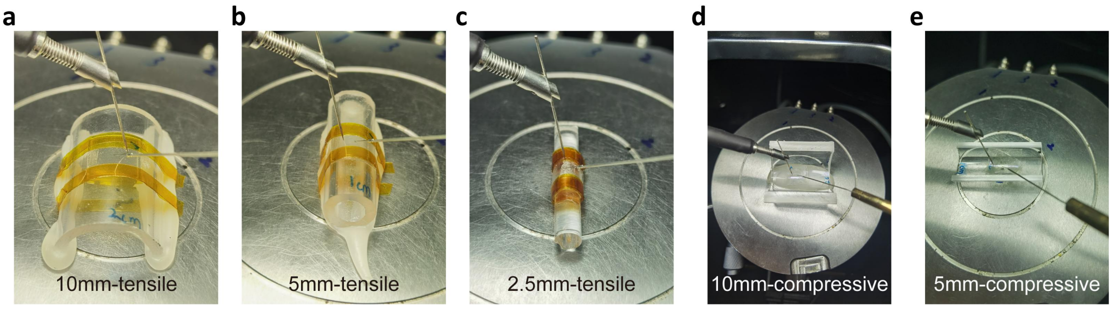

Fig. S6 a-e Bending test molds with different radii as 10 mm of tensile (a) and compressive (d) strain, 5 mm of tensile (b) and compressive (e) strain, and 2.5 mm of tensile strain (c).

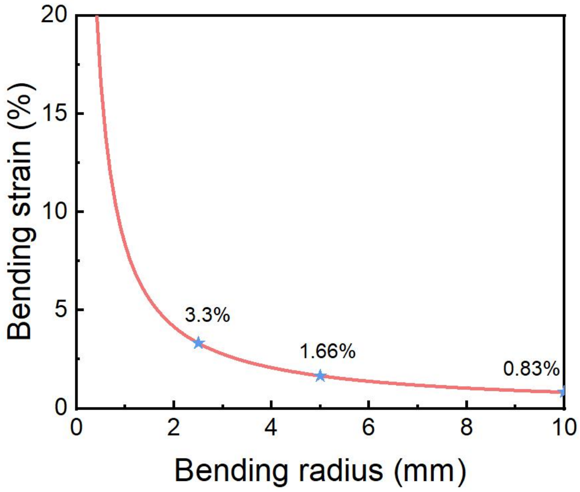

Fig. S7 The variation of strain as a function of the bending radius. When the bending radius is 10 mm, 5mm and 2.5 mm, the maximum tensile/compressive strains are of 0.83%, 1.66% and 3.3%, respectively. The in-plane strain is calculated [1] as $\varepsilon_{ip} = \frac{\frac{Y_1 t_1^2}{2} + Y_2 t_2 \left(t_1 + \frac{t_2}{2}\right) + Y_3 t_3 \left(t_1 + t_2 + \frac{t_3}{2}\right) + \cdots + Y_n t_n \left(t_1 + t_2 + \cdots + t_{n-1} + \frac{t_n}{2}\right)}{R(Y_1 t_1 + Y_2 t_2 + \cdots + Y_n t_n)}$, where Y_n is the Young's modulus at the nth layer, t_n is the thickness at the nth layer (start with the top $PbZrO_3$ layer), R is the bending radius. The Young's modulus of $PbZrO_3$, $SrRuO_3$, ITO and PET layer are 217.37 GPa [2], 161 GPa [3], 89 GPa and 7.5 GPa[1], respectively. The thickness of $PbZrO_3$, $SrRuO_3$, ITO and PET layer are 292 nm, 30 nm, 65 nm and 175 μm, respectively.

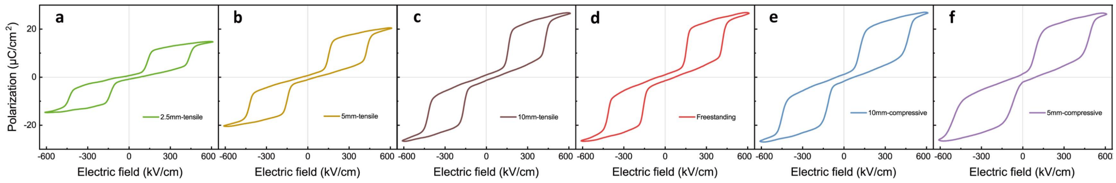

Fig. S8 a-f The P-E hysteresis loops of the freestanding $PbZrO_3$ membranes on the ITO-coated PET substrate (d) and its bending test with different radii as 10 mm of tensile (c) and compressive (e) strain, 5 mm of tensile (b) and compressive (f) strain, and 2.5 mm of tensile strain (a).

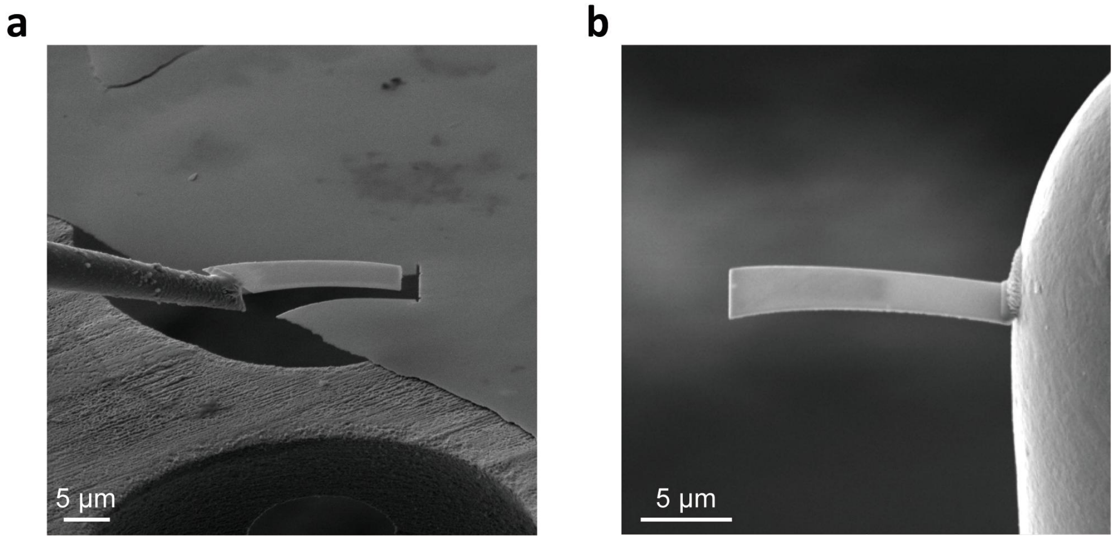

Fig. S9 Morphology of $PbZrO_3$ nanoribbons. **a-b** The SEM images of freestanding $PbZrO_3$ nanoribbons. The freestanding $PbZrO_3$ nanoribbon is cut by a focused ion beam (FIB) with one side fixed to a tip, and this nanoribbon has a size of 14 μm (length) by 2.5μm (width) by 120 nm (thickness).

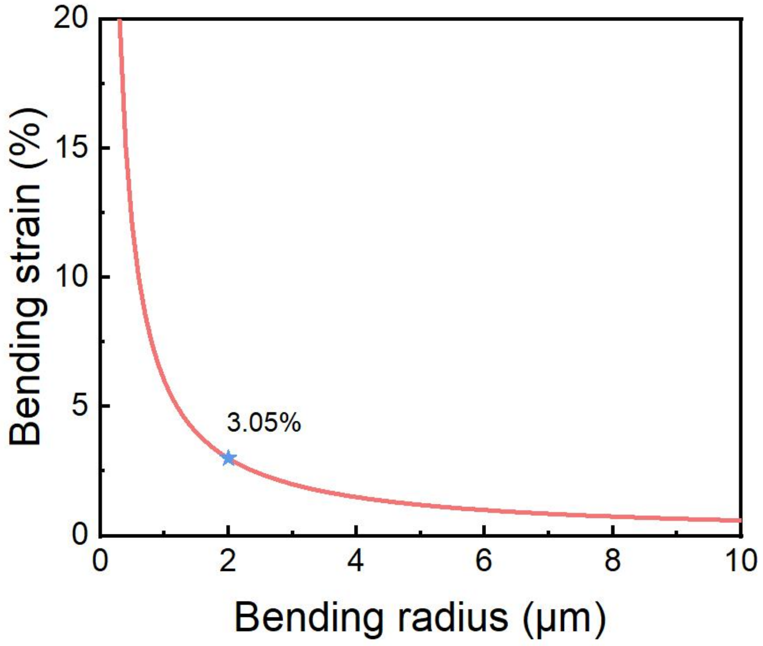

Fig. S10 The variation of strain as a function of the bending radius during *in situ* SEM bending test for a 120 nm freestanding $PbZrO_3$ membrane.

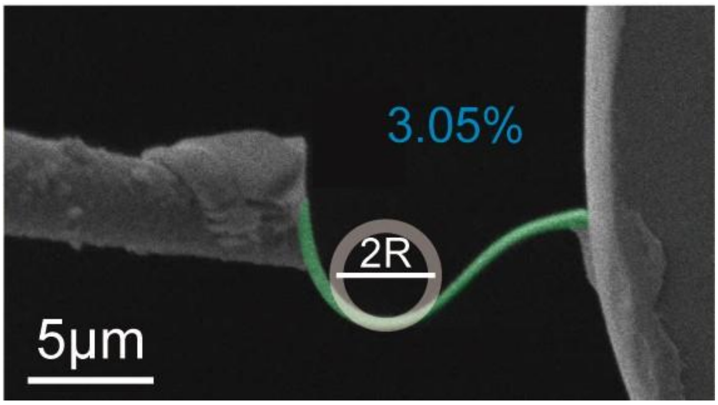

Fig. S11 Estimation of bending strain of the $PbZrO_3$ membrane during the bending process. The scale bar is 5 μm.

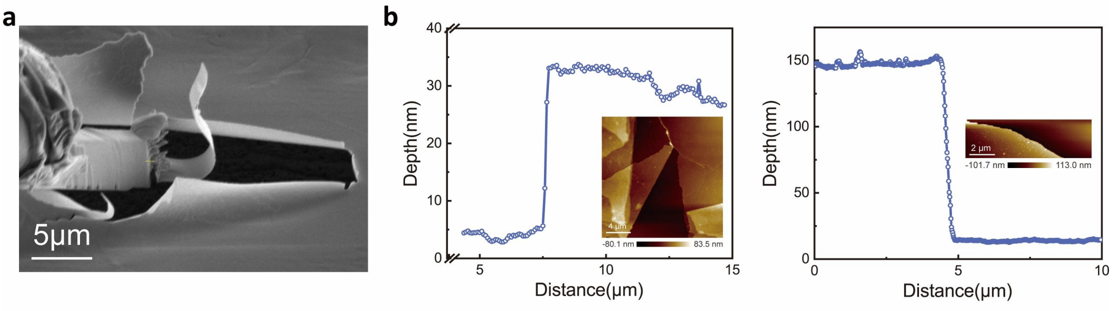

Fig. S12 Morphology and thickness of $PbZrO_3$ nanoribbons. **a** The SEM images of freestanding $PbZrO_3$ nanoribbon with a thickness of 30 nm. **b** The atomic force microscopy (AFM) depth profile of thinner (~30 nm, the left) and thicker (~133 nm, the right) freestanding $PbZrO_3$ membrane transferred on the silicon substrate. The inset presents the surface morphology.

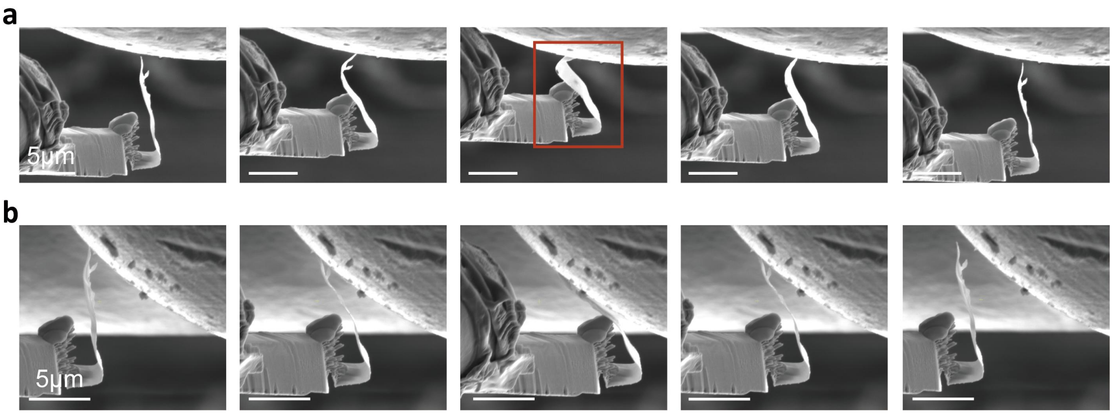

Fig. S13 *In situ* SEM bending test of freestanding $PbZrO_3$ nanoribbon with a thickness of 29 nm. **a-b** The first to fourth columns correspond to the initial, intermediate, maximum and residual bending states during four subsequent bending cycles, respectively. The last column shows the states after the removal of the external load. Scale bars, 5 μm.

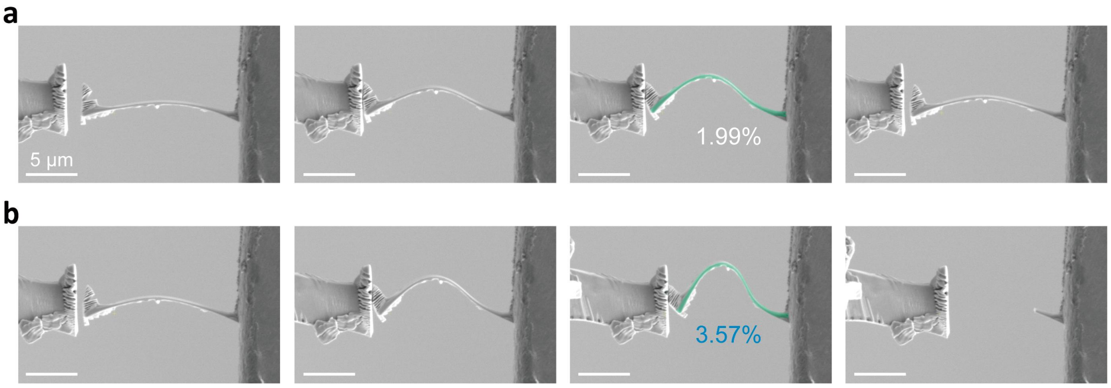

Fig. S14 *In situ* SEM bending test of freestanding $PbZrO_3$ nanoribbon with a thickness of 133 nm. **a-b** The first to fourth columns respectively correspond to the initial, intermediate, maximum and residual bending states during four subsequent bending cycles. Scale bars, 5 μm.

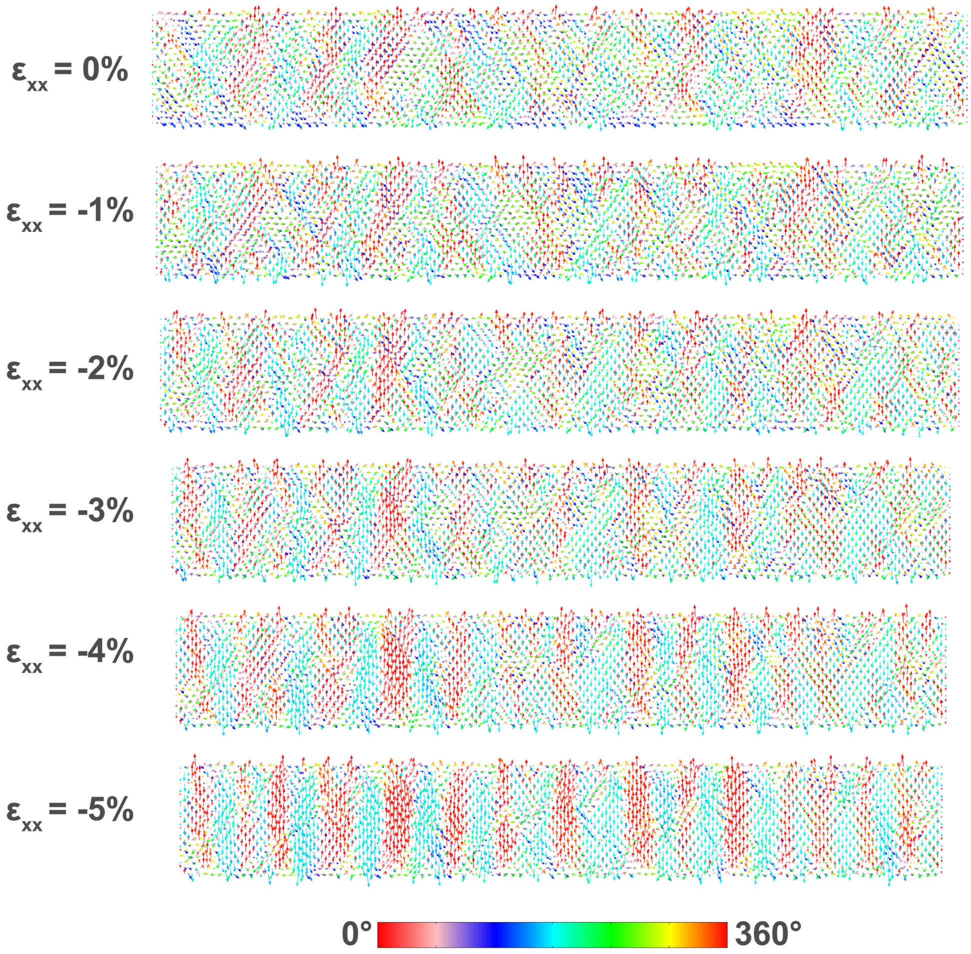

Fig. S15 Typical snapshots of dipole configurations of $PbZrO_3$ under the uniaxial compressive strains ε_{xx} in atomistic simulations.

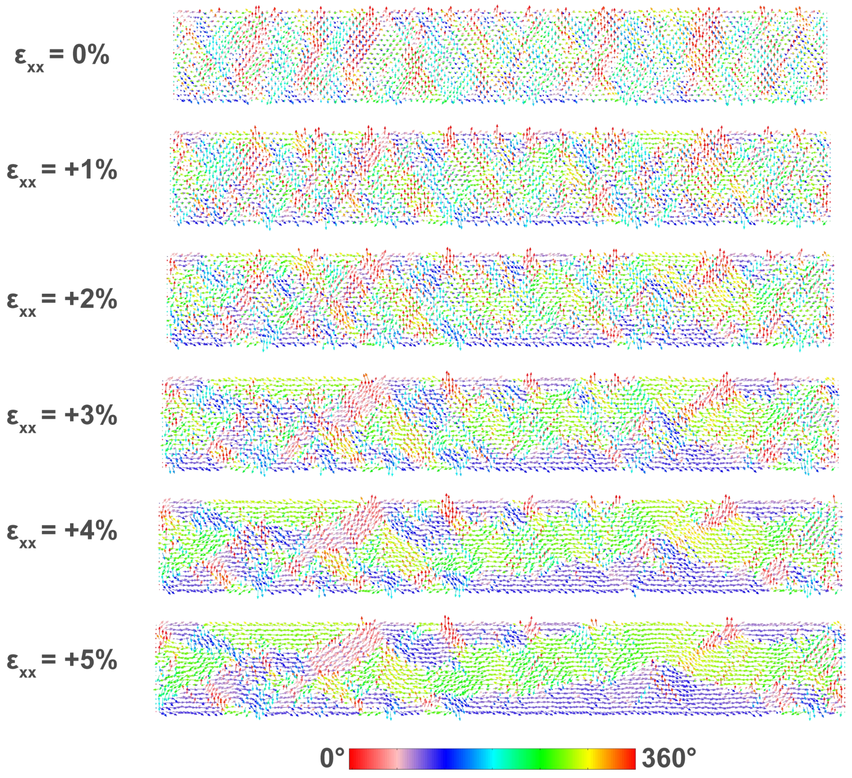

Fig. S16 Typical snapshots of dipole configurations of $PbZrO_3$ under the uniaxial tensile strains ε_{xx} in atomistic simulations.

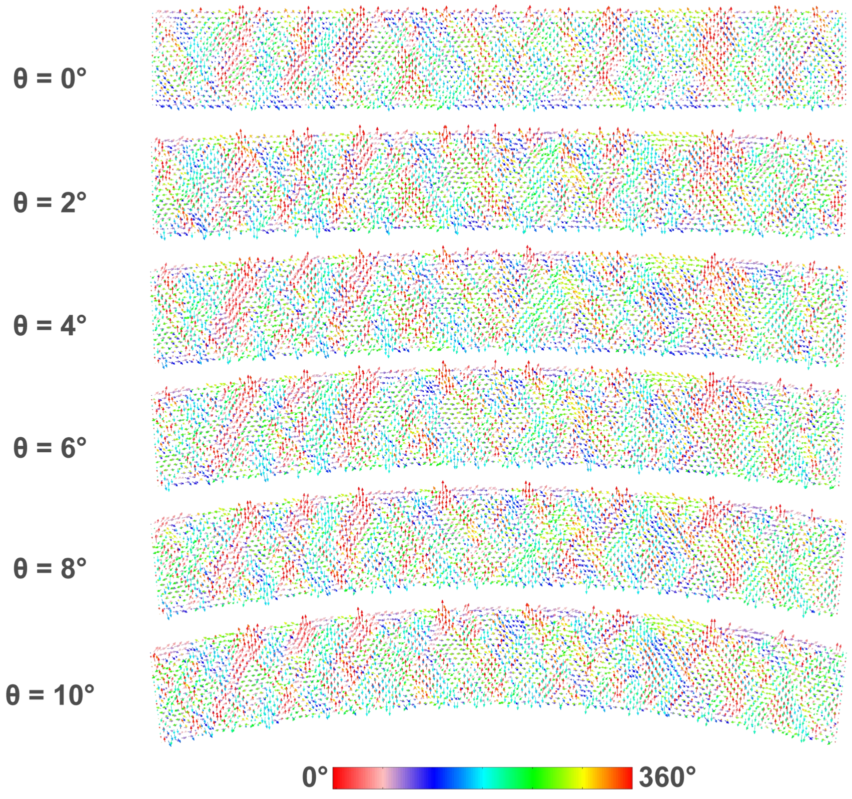

Fig. S17 Typical snapshots of dipole configurations of $PbZrO_3$ during the bending process in atomistic simulations. The θ refer to the bending angle θ in $PbZrO_3$ membrane.

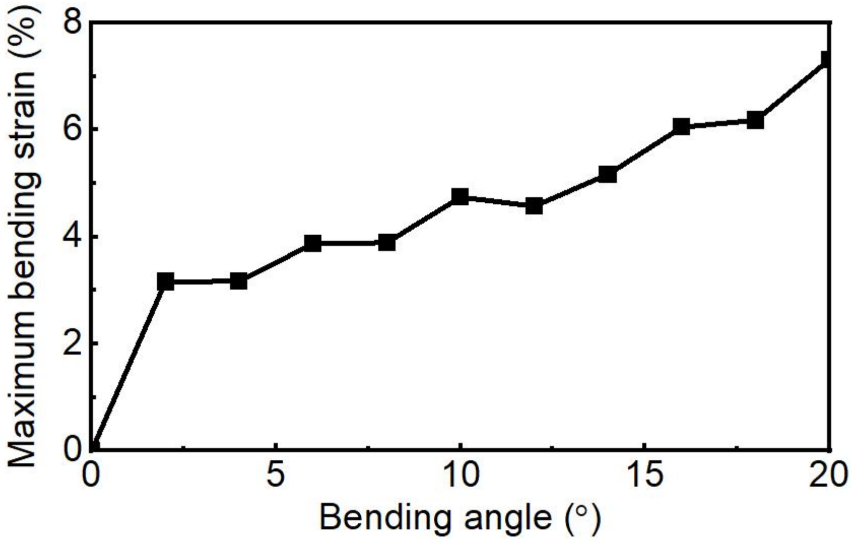

Fig. S18 Atomistic simulations on the maximum bending strain of the $PbZrO_3$ membrane as a function of bending angle.

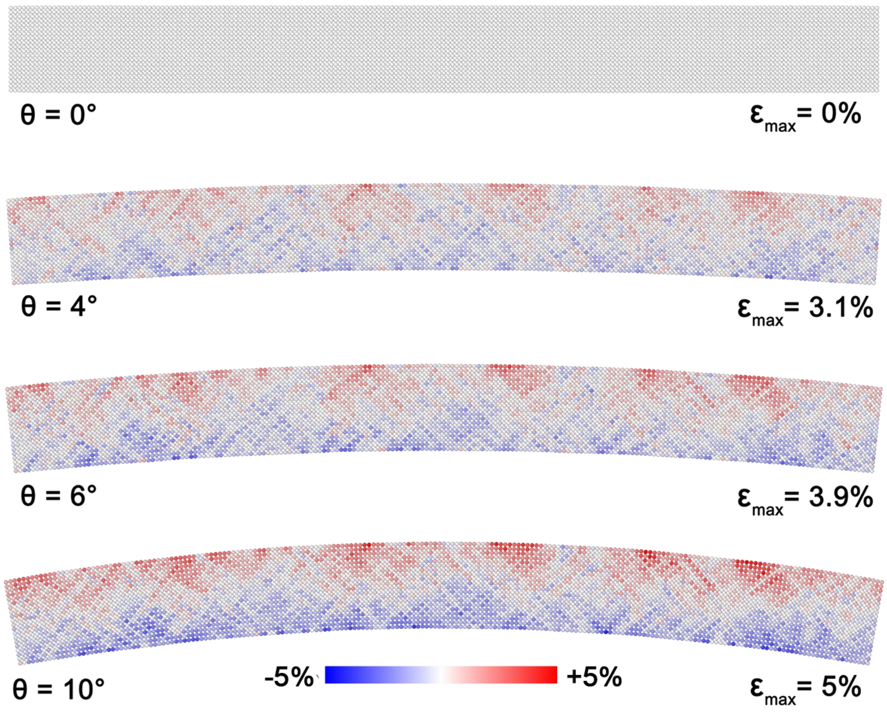

Fig. S19 The strain map for the freestanding single-crystalline $PbZrO_3$ membrane under bending. The color represents the normal strain ε_{xx} along the *x* direction. With the initial stress-free configuration as a reference state, we found ferroelastic domains laying inside the plane are generated in the tensile zones (red patches), while those laying outside of the plane are generated in the compressive zones (blue patches). The ε_{max} and θ refer to the maximum bending strain and the bending angle in $PbZrO_3$ membrane, respectively.

Table S1. The lattice parameter of as-grown and freestanding films. It can be seen that once becoming freestanding, the out-of-plane lattice of $PbZrO_3$ contracts with variations of about -0.23%.

	As-grown	Freestanding
	Out-of-plane [Å]	Out-of-plane [Å]
$PbZrO_3$	8.246 ± 0.004	8.227 ± 0.002

Table S2. Changes of the polarization−field hysteresis loop of antiferroelectric $PbZrO_3$-based films under different bending strains.

Materials	Bending Strain	Change in P_{max}	Change in P_r	Reference
$Pb_{0.97}La_{0.02}Zr_{0.95}Ti_{0.05}O_3$/Ni-foil	±0.22%, ±0.31%	Fluctuates around 85 μC/cm^2	-	4
$Pb_{0.99}Nb_{0.02}(Zr_{0.55}Sn_{0.40}Ti_{0.05})_{0.98}O_3$/$LaNiO_3$/mica	0.14%, 0.21%, 0.28% and 0.42%	Slightly increased	Slightly increased	5
6 mol. % La-doped $PbZrO_3$/$LaNiO_3$/Ni-Cr-foil	~0.5%	Almost unchanged	Almost unchanged	6

Table S3. The radius of curvature (*R*) and corresponding bending strain during the in situ SEM bending test. The bending strain could be obtained by [7] $\varepsilon_{bend} = \frac{t}{2R}$. Here, the thickness of membrane *t* is 120 nm.

	Column 3 (Maximum)		Column 4 (Residual)	
	R (μm)	ε_{bend} (%)	*R* (μm)	ε_{bend} (%)
Fig. 3a	3.93 ± 0.17	1.53 ± 0.07	15.70 ± 1.85	0.38 ± 0.04
Fig. 3b	3.14 ± 0.15	1.91 ± 0.09	11.78 ± 1.60	0.51 ± 0.06
Fig. 3c	2.36 ± 0.21	2.54 ± 0.21	9.81 ± 0.77	0.61 ± 0.04
Fig. 3d	1.97 ± 0.26	3.05 ± 0.36	-	-

Supplementary References

1. Guo R, *et al.* Continuously controllable photoconductance in freestanding $BiFeO_3$ by the macroscopic flexoelectric effect. *Nat. Commun.* **11**, 2571 (2020).

2. Rashid M, Mahmood Q, Babar F, Ramay SM, Mahmood A. Study of mechanical, electronic and optical properties of $PbZrO_3$ and $PbHfO_3$; DFT approach. *Mater. Res. Express.* **6**, 066311 (2019).

3. Yamanaka S, Maekawa T, Muta H, Matsuda T, Kobayashi S-i, Kurosaki K. Thermophysical properties of $SrHfO_3$ and $SrRuO_3$. *J. Solid State Chem.* **177**, 3484-3489 (2004).

4. Li Y*, et al.* Flexible PLZT antiferroelectric film capacitor for energy storage in wide temperature range. J. Alloys Compd. **868**, 159129 (2021).

5. Shen B, Li Y, Sun N, Zhao Y, Hao X. Enhanced energy-storage performance of an all-inorganic flexible bilayer-like antiferroelectric thin film via using electric field engineering. *Nanoscale* **12**, 8958-8968 (2020).

6. Lee HJ*, et al.* Flexible high energy density capacitors using La-doped $PbZrO_3$ anti-ferroelectric thin films. Appl. Phys. Lett. **112**, 092901 (2018).

7. Dong G*, et al.* Super-elastic ferroelectric single-crystal membrane with continuous electric dipole rotation. *Science* **366**, 475-479 (2019).
