# Supplementary material for: Remarkable flexibility in freestanding single-crystalline antiferroelectric PbZrO3 membranes
Source: Nat Commun. 2024 May 24;15:4414. doi: 10.1038/s41467-024-47419-w (PMC11116490; doi:10.1038/s41467-024-47419-w)
Supplement: Supplementary file 3 — Description of Additional Supplementary Files [file 41467_2024_47419_MOESM3_ESM.pdf]

Description of Additional Supplementary Files

Supplementary Movie 1:

In-situ SEM on a $PbZrO_3$ nanobelt (14 μm×2.5 μm×120 nm) during the bending process.
